# Gaps in the ethical governance of pharmaceutical clinical trials in Europe

**DOI:** 10.3389/fmed.2024.1507021

**Published:** 2025-01-07

**Authors:** Rosemarie D. L. C. Bernabe, Shereen A. Dawkins-Cox, Christine C. Gispen-de Wied

**Affiliations:** ^1^Centre for Medical Ethics, Institute of Health and Society, Faculty of Medicine, University of Oslo, Oslo, Norway; ^2^Faculty of Health and Social Sciences, University of South-Eastern Norway, Kongsberg, Norway; ^3^Gispen4Regulatory Science Consultancy, Bilthoven, Netherlands

**Keywords:** ethical governance, clinical trials, research ethics committees, GCP inspections, end-to-end ethics, clinical trial oversight, European Medicines Agency

## Abstract

The ethical governance of pharmaceutical clinical trials in Europe, particularly under Regulation 536/2014, is intended to ensure the safety, rights, and well-being of participants. Despite this regulatory framework, significant gaps in ethical oversight remain. This paper identifies five key deficiencies: (1) European regulations only partially address ethical imperatives set by international guidelines, thereby restricting the ethical mandate of relevant entities; (2) the role of research ethics committees is largely limited to pre-approval activities, reducing continuous oversight during trials; (3) GCP inspectors operate within a narrow scope regarding ethical oversight, which limits their ability to identify a broad range of unethical practices; (4) there is insufficient transparency and collaboration between RECs and regulators, specifically GCP inspectorates, leading to fragmented oversight; and (5) there is minimal integration of ethical findings into the marketing authorization decision process by entities such as clinical assessors and the CHMP. To bridge these gaps, the paper suggests a shift from a prospective ethics review to a comprehensive end-to-end model of ethical governance.

## Introduction

That research activities such as clinical trials must have ethics oversight to ensure the rights, safety, and well-being of research participants is a well-established norm enshrined in various national and international ethics guidelines (1–4). Within the European Union and its associated countries, Regulation 536/2014 on clinical trials on medicinal products for human use (otherwise known as the Clinical Trials Regulation) begins with the statement:

In a clinical trial the rights, safety, dignity and well-being of subjects should be protected and the data generated should be reliable and robust. The interests of the subjects should always take priority over all other interests (5).

Article 4 of this regulation further stipulates that “A clinical trial shall be subject to scientific and ethical review and shall be authorized in accordance with this Regulation. The ethical review shall be performed by an ethics committee in accordance with the law of the Member State concerned” (5). Undoubtedly, research ethics committees (RECs) have a mandate to review and have ethical oversight over these clinical trials. However, ethics oversight is not the sole responsibility of RECs. The document, *Points to Consider on GCP Inspection Findings and the Benefit–Risk Balance*, published by the European Medicines Agency, clearly stipulate that regulators have an ethical mandate as well:

The EU legislation requires not only valid clinical data for the scientific evaluation of the benefit–risk balance, but also ethical conduct of the clinical development program in order to ensure that the rights, safety and well-being of the trial subjects are protected. *GCP inspection findings* - even if not directly influencing the benefit–risk balance – will still be important if they raise serious questions about the rights, safety and well-being of trial subjects and hence the overall ethical conduct of the study. It is an obligation of *clinical assessors, rapporteurs and the CHMP* also to assess the ethics of a clinical development program, and major ethical flaws should have an impact on the final conclusions about approvability of an (marketing authorization) application. Consequently, ethical misconduct could result in rejection of the application (6).

Thus, in the European Union, ethics oversight of clinical trials involves a dual approach, blending both centralized and national processes as stipulated in Regulation (EU) No 536/2014. While the regulation establishes a unified framework for the authorization of clinical trials, it also requires a separate ethical review by national ethics committees. Unlike the assessments done by European regulators such as the CHMP, the ethical review is strictly a national responsibility.

From the above, we could infer that from a governance perspective - and by governance we refer to “the system of values, policies, and institutions by which a society manages its economic, political and social affairs” (7) - the European Union’s position on ethical conduct of drug clinical trials is as follows: human dignity is a value considered inviolable (8) which is put into effect and affects the conduct of clinical trials through the primacy of the rights, safety and well-being of research subjects (5). This is interpreted and governed by European and national institutions and regulators mandated to do so, specifically the national RECs (5), GCP inspectors, clinical assessors, rapporteurs, and the CHMP.

In spite of this position, in earlier publications, we have documented the following:

1. GCP inspectors frequently discover ethically relevant findings (ERFs) during inspections. In fact, around a third of GCP findings were ERFs and that ERFs were present in almost all of the clinical trials with GCP issues (9).
2. The majority of the ERFs were categorized by inspectors as *major* issues (9), i.e., “conditions, practices, or processes that might adversely affect the rights, safety or well-being of the subjects and/or the quality and integrity of data” (10). A tenth of the findings were categorized as *critical* (9), i.e., conditions, practices or processes that “adversely affect the rights, safety or well-being of the subjects and/or the quality and integrity of data” (10).
3. The most common major/critical findings were related to protocol compliance/ protocol issues, patient safety, and professionalism (9). In terms of density, i.e., “the probability that a finding within a category is either critical or major,” the most common issues were monitoring and oversight, protocol compliance/protocol issues, and respect for persons (9). Overall, the most frequent ERFs discovered by inspectors are those that affect the scientific evaluation of the benefit–risk balance of a marketing authorization application (9).
4. In a subsequent publication, we investigated the fate of major and critical ERFs, i.e., whether these in fact affect marketing authorization deliberations (11). This is meant to examine whether major (and, of course, critical) ethical flaws “have an impact on the final conclusions about approvability of an application” (6), as stated in the document, *Points to Consider* (11). The finding was simple and straightforward: none of the major and critical ERFs that were purely ethical in nature (as opposed to issues that were both scientific and ethical) -specifically issues pertaining to informed consent, research ethics committees, and respect for persons – were “explicitly carried over to the joint assessment reports.” This means that at least based on official documents, we cannot find any proof that purely ethical flaws had any impact on the “final conclusions about approvability of an application.”

These findings prompt us to ask: what deficiencies exist in the ethical governance of pharmaceutical clinical trials in Europe? To answer this, we draw on a decade of research. We aim to clarify the implicit aspects in some of our previous publications and, more significantly, integrate our earlier conclusions to address this question.

### Gaps in ethics oversight

This section will outline the responsibilities and duties of entities tasked with ethics oversight as specified in European documentation, and highlight specific deficiencies in ethics governance.

*Regulation 536/2014* Art. 18 states that ethics committees are to evaluate clinical trials “in accordance with international guidelines…,” while *ICH-GCP* Art. 2.1. States that “Clinical trials should be conducted in accordance with the ethical principles that have their origin in the Declaration of Helsinki.” When it comes to what exactly these ethical imperatives say, in a supplement to the publication, *Drug regulators and ethics: which GCP issues are also ethical issues?* (12), we identified the areas covered by various ethics guidelines for research involving human participants, the gist of which is in Table 1.

**Table 1:** List of ethical imperatives according to major international ethical guidelines (recreated from the supplement table in the publication, Drug regulators and ethics: which GCP issues are also ethical issues? (12)).

Ethics imperatives based on international ethics guidelines	
1. Principles	This section discusses the overarching ethical principles that should guide medical research involving human subjects. This includes respecting/prioritizing the welfare of the participants, ensuring justice and fairness, and promoting medical progress responsibly.
2. Research collaboration	This section emphasizes the importance of collaboration with the community where the research takes place. It includes guidelines for community involvement, respecting cultural values, and ensuring fair distribution of benefits.
3. Social value	This section focuses on the importance of research contributing to the well-being of society, particularly the host country. It includes guidelines for responsiveness to health needs, ensuring benefits for the community, and reasonable availability of interventions developed through the research.
4. Scientific validity	This section underscores the need for research to be scientifically sound and credible. It includes guidelines for study design, protocol development, ensuring qualified researchers, and the use of appropriate comparators.
5. Participant selection	This section focuses on the ethical considerations in selecting research participants. This involves ensuring equitable distribution of burdens and benefits, minimizing risks, and justifying the inclusion or exclusion of certain groups.
6. Favorable benefit–risk ratio	This section discusses the need to carefully weigh the potential benefits of the research against the risks to participants. This includes minimizing risks, maximizing benefits, and ensuring that the potential benefits justify any risks involved.
7. Independent review	This section emphasizes the role of Research Ethics Committees (RECs) in ensuring the ethical conduct of research. It outlines the composition, requirements, rights, and responsibilities of RECs, as well as the responsibilities of investigators and sponsors in the review process.
8. Informed consent	This section focuses on the critical importance of obtaining informed consent from research participants. It provides detailed guidelines for ensuring culturally appropriate consent, the information that must be provided, and verifying comprehension and voluntariness. It also discusses consent procedures for specific situations, such as the use of data/specimens and research involving deception.
9. Respect for participants	This section emphasizes the ongoing obligation to treat research participants with respect throughout the research process and beyond. It includes guidelines for ensuring participant safety, protecting privacy and confidentiality, disseminating research results, providing compensation for harm, and ensuring access to medical care.
10. Publication, registration, and regulatory sanctions	This section addresses the importance of transparency and accountability in research. It includes guidelines for disclosing conflicts of interest, publishing accurate results, registering research in public databases, and applying regulatory sanctions in cases of misconduct.
11. Special populations	This section provides specific guidelines for conducting research with vulnerable populations, including persons not able to consent (e.g., minors), pregnant women, and breastfeeding women. It emphasizes additional safeguards, consent procedures, and considerations for these groups.

From the perspective of ethics guidelines, ALL these ethical imperatives ought to apply in all research with human participants, including clinical trials. As such, it would be worthwhile to examine if this assumption is correct.

We saw above that the ethical governance of clinical trials is a joint responsibility of RECs and clinical trial regulators namely GCP inspectors, clinical assessors, rapporteurs, and the CHMP. For the requirement that all the ethical imperatives apply in clinical trials to be true, and since the task of ethics oversight is a shared responsibility, we should be able to identify who has oversight of which ethical imperative.

In the publications, *Drug regulators and ethics* (12) and *The ambivalent place of ethics in European regulatory documents* (13), we partially responded to this question by demonstrating that ICH-GCP and Regulation 536/2014 have articles that (partially) correspond with ethical requirements in guidelines, specifically on the following: Basic principles, Scientific validity, Favorable benefit–risk ratio, Independent review, Informed consent, Respect for participants, Publication and registration, and Special populations. Note that the correspondences between ethics guidelines and European regulations on the specific sections mentioned above are only partial. Ethical guidelines require much more than European regulations do. For example, on the section, Independent review, of the 80 ethical imperatives that fall within it, there were only 18 correspondences, meaning that ICH-GCP and Regulation 536/2014 cover only 22.5% of what ethical guidelines require. Or, the section on Informed consent where ethical guidelines have 98 imperatives, but regulations only correspond to 22 of them. For more information, refer to the article, *Drug regulators and ethics* (12) aside from this partial correspondence, we also notice that European regulations are silent when it comes to ethical imperatives pertaining to Research collaboration, Social value, and Participant selection. The ethics mandate of RECs and the relevant regulators are shaped and constrained by this partiality. *This corresponds to the first gap: European regulations only partially cover ethical imperatives from ethical guidelines, and thus, the ethics mandate of RECs and the relevant regulators would be partial as well*.

This does not yet answer who is responsible for what. Though we do not know of any official document that straightforwardly distinguishes the responsibility of which regulator/ethics committee for which ethical imperative, documents such as ICH-GCP (which is the remit of GCP inspectors) and the European Commission document, *Detailed guidance on the application format and documentation to be submitted in an application for an Ethics Committee opinion on the clinical trial on medicinal products for human use*, could shed some light in terms of some distinction. The latter document outlines the information that RECs in Europe obtain from clinical trial submissions, including information on the following.

- Information about the investigational medicinal product.
- The assessment of expected benefits and risks.
- Justification for the choice of trial participants, particularly when including individuals unable to provide informed consent or other special populations.
- A detailed description of recruitment and informed consent procedures, especially involving participants who are temporarily or permanently incapable of consenting or when a procedure with witnessed consent will be employed.
- An explanation of the plan for additional care of participants after their involvement in the trial has concluded, particularly if it differs from standard care based on the participant’s medical condition.
- A summary of the protocol in the national language (14, 15).
- Assurance of the investigator’s suitability and the quality of the facilities.
- Insurance and indemnity arrangements.
- Compensation details for participants.
- Any significant amendments.
- Safety measures and reporting of adverse events.
- Notification upon completion or early termination of the clinical trial (16).

These sections, and thus RECs, cover the following sections from the list of ethics imperatives: Scientific validity, Participant selection, Favorable benefit–risk ratio, Informed consent, Respect for participants, and Special populations. This list of sections closely resembles the correspondence between ethics guidelines and regulations we identified above. An important observation must be stated, though: RECs in Europe’s tasks are concentrated in the approval stage (14, 15).

While RECs in Europe have a clear role in the initial approval of research, their responsibilities after this stage are far more limited and vary significantly across different jurisdictions. The typical post-approval responsibilities of RECs in Europe are outlined in the publication, *RECs and post-approval activities: a qualitative study on the perspectives of European research ethics committee representatives* (14, 15). Notwithstanding the variations across Europe, RECs may perform at least some of the following activities: review and approval of protocol amendments, receipt and review of annual reports, receipt of serious adverse event reports, receipt of end-of-trial reports, and some limited role in protocol deviations and violations (15). Ethics committees receive and review reports, most often at the level of the secretariat, though some cases require a full-committee review. However, actions required specially for serious adverse events and protocol deviations, and in some instances the review of annual reports, were often relegated to regulatory authorities (15). *This points to the second gap: the function of RECs in Europe continues to be concentrated within the pre-approval stage, thereby severely restricting the mandate of these committees to provide continuous oversight.* We speculate that this constraint could play a role in the previously mentioned frequency with which GCP inspectors discover ethically significant findings after trials.

Transparency in the ethical oversight of clinical trials remains a significant challenge, particularly in the post-approval phase. RECs often lack access to critical information about the ongoing status of clinical trials beyond what they receive as part of required reporting, including findings from inspections and updates on protocol compliance. This is evidenced by the fact that a number of major and critical ethics issues remain and are discovered post-trial, as mentioned above. Additionally, there is very little direct contact or structured communication between RECs and inspectors, which exacerbates the disconnect between these entities. This lack of visibility and collaboration can limit RECs’ ability to provide meaningful oversight beyond the initial approval stage. Enhancing transparency by facilitating information sharing and fostering better communication channels between RECs and inspectors could enable them to address ethical concerns more effectively and in real time, rather than relying solely on retrospective assessments or incomplete data. *This points to the third gap: the insufficient transparency and collaboration between RECs and inspectors, which prevents a cohesive and continuous ethical oversight framework throughout the lifecycle of clinical trials*.

GCP inspectors utilize *ICH-GCP* during inspection, which means that they are mandated to look at all the correspondences we outlined above and to report on divergences. In the publication, *Ethics in clinical trial regulation: ethically relevant issues from EMA inspection reports* (9), we found that inspectors in fact reported on the following:

- protocol compliance or protocol issues
- patient safety
- professionalism and/or qualification issues
- RECs
- informed consent
- monitoring and oversight, and
- respect for persons (9).

These topics correspond to the following sections from the list of ethics imperatives: Scientific validity, Respect for participants, Independent review, and Informed consent. That is even a shorter list compared to the list of ethics imperatives that RECs review. *This points to the fourth gap: GCP inspection has a narrow mandate in terms of ethics oversight, which further limits the type and number of identified unethical conduct*.

There may be multiple factors contributing to this narrow mandate. One factor may be the perception that responsibility for ethics and ethical conduct of research is primarily that of the research ethics committee and the principal investigator. Another factor could be the presumption that ethics is adequately addressed in the prospective review process. A third factor may be an embracing of a trust culture in research, i.e., the idea that once a clinical trial has been cleared at ethics review, then the researcher will in fact conduct the research ethically. While trust is important to maintain good relationships between oversight bodies and researchers, there is a greater moral obligation to protect research participants as emphasized in article 8 of the Declaration of Helsinki. A fourth factor may be insufficient human and other relevant resources necessary for executing the mandate of the regulatory authorities (15). Consequently, only a small percentage of clinical trials are inspected. According to earlier findings, less than 1% of clinical trials submitted to the European Medicines Agency were in fact inspected.

Clinical assessors, rapporteurs, and the CHMP are recipients of inspection reports, and thus are mandated to also look into the flagged ethical issues by the inspectors specifically to weigh the effect of such issues on the approvability of a marketing authorization application, as stated in the document, *Points to consider on GCP inspection findings and the benefit–risk balance* (6): “It is an obligation of clinical assessors, rapporteurs and the CHMP also to assess the ethics of a clinical development program, and major ethical flaws should have an impact on the final conclusions about approvability of an (marketing authorization) application” (6). However, based on the findings in the publication, *Ethics and the marketing authorization of pharmaceuticals: what happens to ethical issues discovered post-trial and pre-marketing authorization?* (11), we have reasons to believe that clinical assessors, rapporteurs, and the CHMP have dispensed of this ethical mandate lightly as none of the major and critical ethical issues flagged by inspectors between 2011 to 2015 were carried over in any of the assessment reports or list of outstanding issues at Day 150 and Day 180 of the centralized procedures for authorizing medicinal products (11). *The foregoing points to the fifth gap: the ethics mandates of clinical assessors, rapporteurs, and the CHMP are not explicitly stated, thus limiting the effects of ethical violations in marketing authorization application deliberations*. It may be pertinent to mention the Clinical Trials Information System (CTIS) (17) and the suggestion to explore the potential for RECs to access essential information, such as inspection reports, thereby enabling them to take a more proactive role in ongoing trial oversight, a point previously raised in *Ethics and Compliance Post-Clinical Trial Approval: the Role of Research Ethics Committees* (18). This opportunity could allow RECs to function independently in assessing ethical compliance, reducing their reliance on other bodies (18), or allow for greater collaboration between the various oversight bodies. The 2024 version of the Declaration of Helsinki has moved in this direction as Article 23 reads:

The committee must have the right to monitor, recommend changes to, withdraw approval for, and suspend ongoing research. Where monitoring is required, the researcher must provide information to the committee and/or competent data and safety monitoring entity, especially about any serious adverse events. No amendment to the protocol may be made without consideration and approval by the committee. After the end of the research, the researchers must submit a final report to the committee containing a summary of the findings and conclusions (1).

Given that the Regulations permit member states to structure their ethics review processes differently, with some countries already being more active in overseeing ongoing trials, there is a clear opportunity to call for greater harmonization across Europe.

## Discussion

The ethical governance of pharmaceutical clinical trials in Europe demonstrates significant challenges, despite the overarching regulatory framework provided by Regulation 536/2014. This discussion explores the broader implications of these gaps for ethical oversight, regulatory practice, and participant protection.

### Addressing the identified gaps

The foregoing highlights five central gaps in ethical governance: partial alignment of European regulations with international ethical guidelines, limited post-approval oversight by RECs, narrow scopes for GCP inspections, insufficient transparency and collaboration between RECs and health inspectorates, and minimal integration of ethical considerations into the marketing authorization process. These gaps collectively weaken the ethical oversight framework, leaving room for potential violations that could undermine participant safety and trust, as evidenced by the persistence of ethically relevant findings in clinical trials discovered post-trial and during inspection.

#### Partial ethical alignment

The ethical imperatives of international guidelines, such as the CIOMS Guidelines and the Declaration of Helsinki, emphasize comprehensive oversight encompassing all stages of clinical trials. However, European regulations fail to fully incorporate these principles, particularly in areas like research collaboration, social value, and participant selection. This partiality restricts the ethical mandate of the relevant regulatory bodies, necessitating urgent updates to align regulations with globally recognized ethical standards.

#### Limited REC oversight post-approval

RECs play a pivotal role during the pre-approval stage but lack substantial mandates for ongoing oversight. This disconnect contributes to ethical oversights uncovered during inspections, highlighting the need for an end-to-end ethics review model. Incorporating a lifecycle approach would empower RECs to monitor and address ethical concerns throughout the trial process, mitigating risks to participants.

#### Narrow scope of GCP inspections

While GCP inspectors are equipped to identify some ERFs, their focus remains constrained. The reliance on trust in researchers’ compliance and the limited percentage of trials inspected exacerbate this issue. Expanding the scope and frequency of inspections and/or improving cooperation between RECs and clinical trial regulators such as the GCP inspectors, could ensure more robust ethical compliance.

#### Insufficient transparency and collaboration

Transparency in ethical oversight is a critical yet under-addressed challenge. There is little direct communication between RECs and clinical trial regulators such as health inspectorates, and RECs often lack access to critical information such as inspection findings. Enhanced collaboration, including mechanisms for regular information sharing, could bridge these transparency gaps without duplicating efforts. Leveraging tools like the Clinical Trials Information System (CTIS) could facilitate centralized tracking and enable RECs to maintain an informed role in ongoing oversight, ensuring ethical issues are dynamically addressed throughout the trial lifecycle.

#### Ethics in regulatory decision-making

Despite the acknowledgment of ethical oversight as a crucial aspect of the benefit–risk assessment for marketing authorization, evidence suggests that ethical violations are rarely integrated into final regulatory decisions. This disconnect undermines the ethical framework and the credibility of regulatory processes. Regulatory bodies must establish clearer guidelines and accountability mechanisms to ensure that ethical findings substantively influence marketing approval outcomes.

### Toward an end-to-end ethical governance model

The findings underscore the need for a paradigmatic shift from prospective ethics review to an integrated, end-to-end governance framework. Such a model would:

Enhance regulatory collaboration: Strengthening the interaction between RECs, GCP inspectors, and regulatory agencies to share findings and address ethical concerns dynamically. Mechanisms for regular information sharing, such as providing RECs access to inspection findings and trial progress updates via platforms like CTIS, could foster transparency and collaboration.

Leverage technological tools: Platforms like CTIS present opportunities for centralized tracking and reporting of ethical compliance, reducing reliance on fragmented updates.

Harmonize practices across member states: A unified approach would reduce disparities in ethical governance and enhance accountability.

### Limitations and future directions

While this manuscript provides a detailed examination of ethical governance gaps, further research is needed to explore specific solutions and their practical implementation. Note, too, that this manuscript is limited by the fact that we included European but not national regulations, nor did we look at differences and nuances in ethics deliberations among the European member states. Comparative analyses of other regions with stringent ethical governance frameworks could offer valuable insights.

## Conclusion

A robust ethical oversight system is foundational to the integrity of clinical trials and the preservation of inalienable rights of research participants. By addressing the identified gaps—partial ethical alignment, limited post-approval oversight, narrow inspection mandates, insufficient transparency and collaboration, and minimal integration of ethics into regulatory decisions—Europe can strengthen the ethical framework of clinical trials.

Adopting an end-to-end model of ethical governance will ensure that participant rights, safety, and well-being remain central to clinical research, while fostering public trust and innovation. By proactively addressing these challenges, Europe has the opportunity to position itself as a global leader in ethically robust clinical trials.

## Data Availability

The original contributions presented in the study are included in the article/supplementary material, further inquiries can be directed to the corresponding author.
